# Effects of upper thoracic Mulligan mobilization on pain, range of motion and function in patients with mechanical neck pain: A randomized placebo-controlled trial

**DOI:** 10.1371/journal.pone.0311206

**Published:** 2024-10-28

**Authors:** Ramazan Cevik, Omer Osman Pala

**Affiliations:** 1 Mengucek Gazi Education and Research Hospital, Erzincan Binali Yıldırım University, Erzincan, Turkey; 2 Department of Physiotherapy and Rehabilitation, Faculty of Health Sciences, Bolu Abant İzzet Baysal University, Bolu, Turkey; Adnan Menderes Universitesi, TÜRKIYE

## Abstract

**Objective:**

This study aimed to assess the impact of Mulligan Reverse Natural Apophyseal Glides (RNAGS) applied to the upper thoracic region on pain, movement limitation, and function in individuals with mechanical neck pain.

**Methods:**

Conducted between January 2021 and May 2021, the study involved 69 participants randomly assigned to a sham group, physiotherapy group, and Mulligan group. A two-week, 11-session treatment program was administered with shared physiotherapy interventions and stretching exercises to all groups. The Mulligan group received additional mobilization with the Mulligan RNAGS technique. The sham group received sub-standard Mulligan mobilization. Outcome measures included Range of Motion (ROM), Visual Analogue Scale (VAS), and Neck Disability Index (NDI).

**Results:**

No baseline differences were found in measurements among the groups. After the intervention, all groups showed increased ROM and decreased VAS and NDI scores (p<0.001). The Mulligan group exhibited significantly greater improvement in VAS, ROM, and NDI compared to other groups (p<0.001). The sham group demonstrated greater improvement in NDI and extension ROM compared to the physiotherapy group (p<0.001).

**Conclusion:**

Mulligan RNAGS technique in the upper thoracic region proved beneficial for pain relief, range of motion, and functionality in mechanical neck pain. Long-term effects warrant further exploration through population-based studies.

## Introduction

Neck pain is an important public health problem [1], especially since it ranks 11th among diseases that impact disability-adjusted life years [2, 3]. According to an international systematic review by Hoy et al., the annual incidence of activity-limiting neck pain ranges from 10.4–21.3%, with a prevalence of 0.4–86.8%. While 33–65% of cases recover from neck pain within one year [4], symptoms may persist to some degree or relapse regardless of treatment [5]. There are various options for treatment, and the primary approach is evidently targeting potential underlying conditions, if any. However, neck pain involves numerous pathologies in different combinations, including those affecting neurovascular, soft tissue and musculoskeletal structures [6]. These pathologies have been shown to manifest due to an array of factors, such as long-term stress, anxiety and depression, biological risk factors, autoimmune diseases, metabolic alterations, and sociodemographic characteristics [7, 8]. Therefore, therapeutic approaches largely focus on the relief of pain and other potential symptoms.

In the absence of urgent care needs, treatment of neck pain is planned based on patients’ pain level and function. Many patients improve over time with non-surgical care [9]. Conservative options include muscle relaxants, non-steroidal anti-inflammatory drugs, and physical therapy methods such as exercise, massage, acupuncture, yoga and spinal manipulation [5]. Various forms of manipulation and mobilization are widely used [6, 10, 11]. Direct hand contact during manual therapy provides sensory input to the body, stimulates pain-related central nervous system areas, and increases the release of compounds that have analgesic and anti-inflammatory effects. Both forms of manual therapy, namely mobilization and manipulation, appear to create similar results in terms of pain treatment due to the similarities between supraspinal and spinal mechanisms of action that are achieved with these two methods [12, 13]. There are different concepts in manual therapy, such as the Mckenzi, Kalterrnborn, Mulligan and Maitland methods [14], which are reported to be superior to other interventions as determined by pain and function evaluations in chronic non-specific neck pain [15]. The Mulligan mobilization technique aims to correct neck posture by stimulating mechanoreceptors and proprioceptors around relevant joints [11]. While research on this topic continues to expand, evidence is still scarce regarding the selection of the optimal manual therapy technique to be used in the spine region based on clinical findings [16]. In addition, thoracic spine mobilization may be comparable to sham therapy for neck pain, but the evidence is limited. Touch in manual therapy can have significant effects on patients, including physiological and psychological effects through stimulation of c-tactile afferents [17]. Therefore, the study was designed with three groups to measure this effect as well. We sought to assess the efficacy of physiotherapy interventions (stretching exercise, hot-pack application, transcutaneous electrical nerve stimulation [TENS], ultrasonography) and the upper thoracic Mulligan Reverse Natural Apophyseal Glides (RNAGS) mobilization technique, in addressing pain, range of motion (ROM), and functionality for individuals experiencing mechanical neck pain in the upper thoracic region.

## Methods

This placebo-controlled randomized study was conducted between January 2021 and May 2021 at Erzincan Binali Yıldırım University Mengücek Gazi Training and Research Hospital, Department of Physical Therapy and Rehabilitation.

Ethics committee approval was obtained from the Clinical Research Ethics Committee of Sivas Cumhuriyet University (Decisipn date: 16.12.2020, decision no: 2020-12/13) and the study was carried out in accordance with the Declaration of Helsinki and comparable ethical guidelines. The study was registered at www.clinicaltrials.gov under NCT06200038. After each of the individuals in the research group was informed in detail about the purpose and scope of the study, those who agreed to participate in the study were asked to read the Informed Consent Form and their signatures were obtained.

### Enrollment of the study group

G*Power 3.1 was used for a priori sample size estimation with an effect size of 0.25, an alpha level of 0.05, and 1-β = 90%. The effect size was obtained from a study which randomized clinic trial using Neck Disability Index (NDI) as primer outcome [18]. According to the results of the sample size estimation, 22 participants were needed for each group to detect a significant group-time interaction We only considered the inclusion of patients who were 18 years of age or older, had a diagnosis of mechanical neck pain for at least three months, and agreed to participate in the study. We excluded patients with a history of spinal surgery, osteoporosis, osteopenia (T-score < -1), rheumatoid arthritis, ankylosing spondylitis, presence of cord compression, vertebrobasilar artery insufficiency, severe radiculopathy, spinal cord tumors, history of Whiplash injury, history of trauma in the cervical/thoracic region and pregnancy.

Of the 75 patients who were eligible for inclusion, four were excluded based on exclusion criteria, 2 were excluded because they did not accept to participate in the study, and thus, a final total of 69 individuals were enrolled and analyzed.

### Randomization process and determination of groups

**Fig 1:**
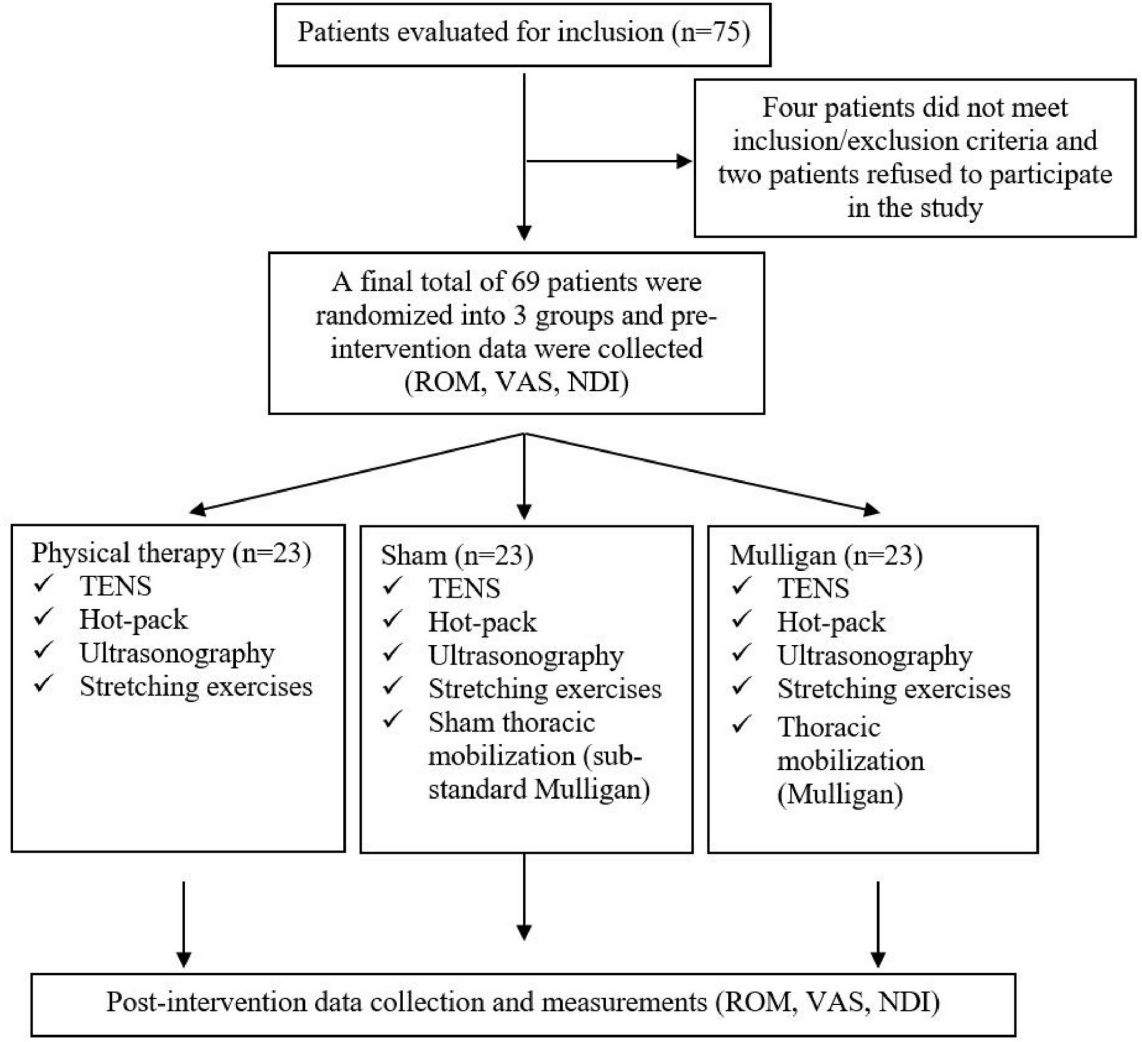
Flowchart of the study.

The individuals in the study group were randomly divided into three groups: sham (n-23), physical therapy (n = 23) and Mulligan (n = 23). The process of random allocation was performed with the online randomization tool "Graphpad". The flow chart describing study design is shown in **Fig 1**.

### Measurements before and after the intervention

The sociodemographic and clinical characteristics of individuals who attended their scheduled inclusion visit were questioned and the data obtained during this process were recorded. Afterwards, detailed physical examinations were performed and ROM, Visual Analogue Scale (VAS), NDI were measured to calculate pre-intervention scores. The measurements required to determine the post-intervention scores were performed immediately after the 11th session at the end of the two-week intervention.

### Range of Motion (ROM)

The DrGoniometer (iOS) mobile application was used for cervical ROM measurements [19]. Active flexion, extension, right/left lateral flexion and rotation of the cervical region were measured in the sitting position.

### Visual Analogue Scale (VAS)

VAS was used to assess the severity of mechanical neck pain. Individuals were asked to mark the pain they felt on a 10 cm long VAS scale. On the VAS, 0 indicates that the pain is not felt at all, 10 indicates unbearable pain, and the pain level increases as the score increases [20].

### Neck Disability Index (NDI)

In the study, the degree of disability in the neck region was evaluated with the Neck Disability Index (NDI). This index, which has proven reliability and validity, contains 10 items. Seven items are related to activities of daily living, two are related to pain intensity and one is related to concentration. The response to each item is scored between 0 (no limitation) and 5 (maximum limitation) and the final NDI score is obtained by summing each score. Higher NDI score means greater degree of neck disability, and the level of neck disability can be categorized according to the score [21].

### Interventions

Patients underwent an 11-session treatment program over a two-week period—daily for the initial week and every other day for the subsequent week. The same physiotherapy applications and stretching exercises (upper trapezius and levator scapula) were applied to the neck region in all three groups [22]. Hot pack was applied to the neck and upper trapezius region for 20 minutes to increase circulation and relax the soft tissue by vasodilatation effect in the vessels [23, 24]. Conventional TENS was applied to the neck area with 2 channels and 4 electrodes for 20 min (current passage time: 50–100 microseconds, frequency: 0–120 Hz) with a mild tingling sensation at a level that does not cause discomfort [23, 25]. Ultrasonography was performed for 8 minutes with an intensity of 1.5 w/cm2 and a frequency of 1 MHz [25]. Stretching exercises were applied to the upper part of the trapezius and levator scapula muscles for 15–30 seconds for 10 repetitions with the help of a physiotherapist. In the Mulligan group, in addition to these applications, mobilization was applied to the upper thoracic segments with the RNAGS technique. Briefly, while the patient was sitting, the transverse process of the lower vertebra of the facet joint was pushed along the treatment plane cranio-ventrally [26]. The application was performed with one hand (thumb in extension, other fingers flexed) that was used to perform the pushing maneuver on the transverse processes, while the other arm was used to gently grasp the head of the patient and recline the neck against the body for stabilization. Each treatment consisted of 3 sets of 10 repetitions with 20-second intervals between sets. In the sham group, sham mobilization was applied to the segments where Mulligan mobilization was performed, where the direction of pushing and the pushing force were different (sub-standard Mulligan mobilization). All applications were performed by a physiotherapist with 10 years of experience who was certified in the Mulligan mobilization technique.

### Statistical analysis

All analyses were conducted using IBM SPSS Statistics for Windows, Version 25.0 (IBM Corp., Armonk, NY, USA). The Shapiro-Wilk test was employed for normality checks. Continuous variables are presented as mean ± standard deviation or median (1st quartile - 3rd quartile), depending on the normality of distribution. Categorical variables are expressed as frequency (percentage).

Between-group analyses for normally distributed variables were carried out using the one-way analysis of variances (ANOVA). For categorical variables, between-group analyses employed the chi-square test or Fisher-Freeman-Halton test. Normally distributed repeated measurements were subjected to two-way repeated measures analysis of variances (ANOVA), while non-normally distributed repeated measurements were assessed using the Wilcoxon signed ranks test. Between-group comparisons for these variables were performed with the Kruskal-Wallis test. Pairwise comparisons were adjusted using the Bonferroni correction method. Statistical significance was set at a two-tailed p-value of less than 0.05.

## Results

Thirty-four (49.28%) of the patients were female, 35 (50.72%) were male, and the mean age was 38.46 ± 9.59 (range 21–57) years. There were 23 patients in each group and all groups were similar in terms of age (p = 0.862) and gender distribution (p = 0.793). There were no differences between the groups in terms of height (p = 0.463), weight (p = 0.797), body mass index (p = 0.991), educational status (p = 0.453), marital status (p = 0.814) and dominant side (p = 0.768) (**Table 1**).

**Table 1:** Summary of demographics, range of motions and assessment scores with regard to treatment groups.

	Groups			
	Sham (n = 23)	Physical therapy (n = 23)	Mulligan (n = 23)	p (between groups)
Age	38.43 ± 9.74	39.26 ± 11.52	37.70 ± 7.47	0.862
Sex				
Female	10 (43.48%)	12 (52.17%)	12 (52.17%)	0.793
Male	13 (56.52%)	11 (47.83%)	11 (47.83%)	
Height, cm	168.26 ± 7.68	168.39 ± 7.40	166.04 ± 6.36	0.463
Weight, kg	74.30 ± 13.27	74.04 ± 12.21	72.09 ± 11.00	0.797
Body mass index, kg/m^2^	26.08 ± 3.19	25.97 ± 2.86	26.06 ± 3.11	0.991
Education status				
Primary school	1 (4.35%)	1 (4.35%)	2 (8.70%)	0.453
Secondary school	5 (21.74%)	3 (13.04%)	1 (4.35%)	
High school	5 (21.74%)	8 (34.78%)	11 (47.83%)	
University	11 (47.83%)	11 (47.83%)	9 (39.13%)	
Postgraduate	1 (4.35%)	0 (0.00%)	0 (0.00%)	
Marital status				
Single	8 (34.78%)	7 (30.43%)	6 (26.09%)	0.814
Married	15 (65.22%)	16 (69.57%)	17 (73.91%)	
Dominant side				
Right	22 (95.65%)	23 (100.00%)	21 (91.30%)	0.768
Left	1 (4.35%)	0 (0.00%)	2 (8.70%)	
Visual Analogue Scale score				
Before	7.08 ± 1.08	7.54 ± 1.04	7.57 ± 1.17	0.240
After	3.89 ± 1.27	5.26 ± 1.25*	2.71 ± 1.10*^#^	<0.001
p (within groups)	<0.001	<0.001	<0.001	
Change ^(1)^	-3.19 ± 1.28	-2.28 ± 1.24	-4.86 ± 1.87*^#^	<0.001
Flexion ROM				
Before	42 (38.1–52)	40 (37.5–48)	43 (38–52)	0.845
After	49.7 (42.7–57)	45 (40.4–52)	55 (49–70)^#^	0.001
p (within groups)	<0.001	<0.001	<0.001	
Change ^(1)^	6 (5–7)	3.9 (2–5)	15 (10.3–17)*^#^	<0.001
Extension ROM				
Before	45.4 (42.6–52)	46 (40.1–54)	42 (39.4–49)	0.164
After	55 (51–59)	49 (42.4–54)	61 (57–69)^#^	<0.001
p (within groups)	<0.001	<0.001	<0.001	
Change ^(1)^	6 (5.6–9)	2.4 (1–4.8)*	17.3 (15–20)*^#^	<0.001
Right rotation ROM				
Before	53 (49.8–58)	51.3 (48.1–58)	51 (47–60)	0.617
After	62 (58–65)	57 (52–64.7)	72.4 (69–76.1)*^#^	<0.001
p (within groups)	<0.001	<0.001	<0.001	
Change ^(1)^	6 (5–8.6)	3.5 (2.7–6.7)	18 (15–23)*^#^	<0.001
Left rotation ROM				
Before	52 (51–59.8)	52 (46–59)	52 (48.9–61)	0.652
After	61 (57–64)	56 (53–63)	72 (68–77)*^#^	<0.001
p (within groups)	<0.001	<0.001	<0.001	
Change ^(1)^	7 (4.2–8)	5 (2.9–7.5)	18 (14.9–22.9)*^#^	<0.001
Right lateral flexion ROM				
Before	23 (21–24)	22 (20–24)	22 (20–24)	0.521
After	26 (24–28)	23.4 (22–25)	33 (29–38)*^#^	<0.001
p (within groups)	<0.001	<0.001	<0.001	
Change ^(1)^	3.7 (1.3–4.7)	1.1 (1–3)	10 (7–13.4)*^#^	<0.001
Left lateral flexion ROM				
Before	23.14 ± 2.67	22.65 ± 2.63	22.92 ± 2.45	0.815
After	26.55 ± 3.37	24.90 ± 3.05	32.27 ± 4.83*^#^	<0.001
p (within groups)	<0.001	0.002	<0.001	
Change ^(1)^	3.41 ± 2.57	2.25 ± 2.42	9.35 ± 4.51*^#^	<0.001
Neck Disability Index score				
Before	20.43 ± 3.22	20.96 ± 3.27	22.57 ± 3.53	0.086
After	14.74 ± 3.40	17.39 ± 3.31*	10.26 ± 1.91*^#^	<0.001
p (within groups)	<0.001	<0.001	<0.001	
Change ^(1)^	-5.70 ± 2.65	-3.57 ± 1.34*	-12.30 ± 3.20*^#^	<0.001

Data are given as mean ± standard deviation or median (1st quartile - 3rd quartile) for continuous variables according to normality of distribution and as frequency (percentage) for categorical variables. ROM: Range of motion. (1) Positive values represent increase and negative values represent decrease.

*: Significantly different from "Sham"

#: Significantly different from "Physical therapy".

There was no difference between the groups in terms of VAS score (p = 0.240), flexion ROM (p = 0.845), extension ROM (p = 0.164), right rotation ROM (p = 0.617), left rotation ROM (p = 0.652), right lateral flexion ROM (p = 0.521), left lateral flexion ROM (p = 0.815) and NDI score (p = 0.086) values before intervention (**Table 1**).

In all groups, ROM values in all directions increased significantly and VAS and NDI scores decreased significantly after the intervention compared to pre-intervention values (p<0.001 for each measurement in each group, except for p = 0.002 for left lateral flexion ROM value in the physical therapy group) (**Table 1**).

**Fig 2:**
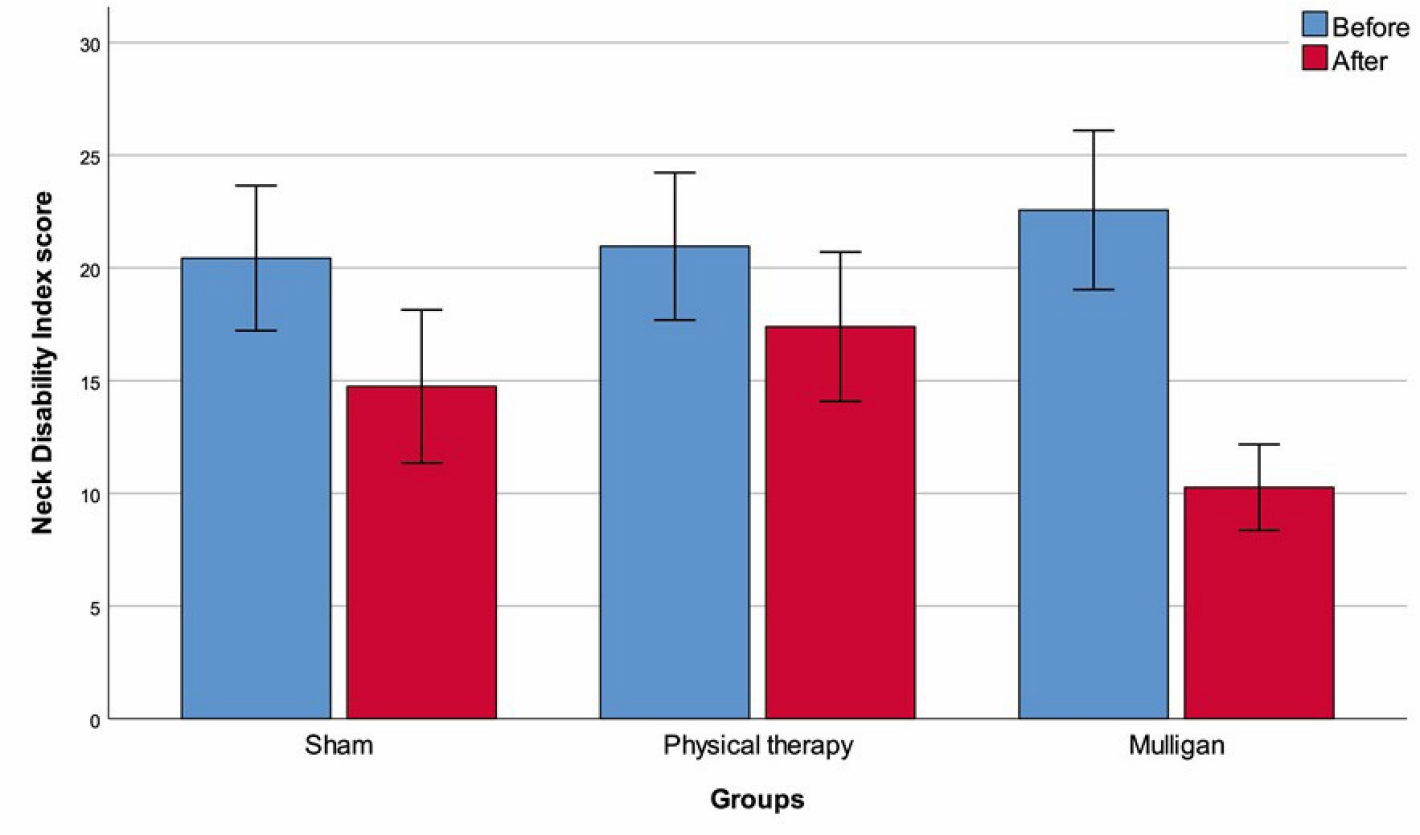
Neck Disability Index scores (mean ± standard deviation) with regard to groups.

In terms of the amount of change in VAS, ROM in all directions and NDI score with intervention, the values measured in the Mulligan group were significantly higher than both the sham group and the physical therapy group (p<0.001 for each measurement parameter). Interestingly, the sham group demonstrated significantly greater improvement in NDI score and extension ROM values compared to the physical therapy only group (p<0.001, **Table 1 and Fig 2**).

## Discussion

The comparison of these treatment modalities in our study demonstrated that Mulligan recipients had superior outcomes compared to the sham and physical therapy groups, as revealed by greater improvements in VAS, ROM in all directions, and NDI scores. Despite this clear advantage, it is worth noting that the sham group (in which patients received substandard Mulligan therapy) also had notable improvements in NDI scores and extension ROM. As a matter of fact, the results showed that these improvements were greater compared to the recipients of only physical therapy, which suggests that manual mobilization appears to have benefits even with substandard utilization. This may be due to the beneficial stimulation of mechanoreceptors as a result of physical contact in both the sham and Mulligan therapy groups, potentially activating similar pathways that affect cortex stimulation. Nonetheless, our findings clearly exemplify the efficacy of the standard RNAGS Mulligan technique in addressing pain, ROM, and functionality in individuals with mechanical neck pain.

The anatomical, biomechanical and neurological characteristics of the cervical and the thoracic regions are among the factors that contribute to neck pain and other spine-related pain [13, 27]. It has been reported that the differences in the mobility of the upper/lower cervical regions and the thoracic region could be effective in cervical musculoskeletal problems [22, 28]. It is therefore advisable to perform examination of the thoracic region as well as the cervical region in persons with mechanical neck pain. Dysfunction in the lower cervical region may cause pain in the thoracic region, which could result in pain-induced movement limitations in the thoracic region, thereby creating a vicious cycle. Indeed, previous studies have suggested that the treatment of problems in the cervical region are reliant upon biomechanical characteristics and correct alignment of the thoracic region [13, 29]. There are reports indicating that hands-on therapy applied near the painful spot, but not directly on it, can actually lessen pain and muscle tightness, possibly through activation of different pathways [30]. Manipulating the thoracic spine seems to help lessen perceived neck pain in people with mechanical neck pain, at least in the short term. Since there are worries about the potential risks of directly manipulating the cervical spine, working on the thoracic spine could be a reasonable option [31]. Few studies have evaluated the effects of thoracic mobilization for neck pain; however, thoracic manipulation (alone or in combination with other therapies) has been reported to improve pain and function in patients with neck pain [10]. In a study with people diagnosed with forward head posture, the findings revealed that mobilizing the thoracic spine led to more immediate improvements in cervical extension, neck pain, and neck disability compared to mobilizing the upper cervical spine and doing stabilization exercises [32]. Several randomized controlled trials have all but proven the added benefits obtained with the use of manual therapy methods (including thoracic) in patients with neck pain [33]. Mobilization has also been shown to create particular benefits in improving ROM among patients receiving strengthening and stretching exercises [34]. It is also crucial to note that expanding the area of administration (thoracic + cervical) has been found to also increase functionality [35]. In our current study, we discovered that using the Mulligan RNAGS technique for mobilization in the upper thoracic region of individuals with mechanical neck pain provided more advantages compared to other physical therapy approaches and placebo mobilization. This was evident in terms of pain reduction, decreased disability, and improved range of motion in the neck joint. Importantly, no adverse effects were observed. In managing neck pain through physical therapy, it appears that concentrating solely on the cervical region would limit the possible improvements that can be obtained with manual therapy. Incorporating mobilization with the Mulligan RNAGS technique in the upper thoracic region, along with traditional physical therapy methods for the cervical region, appears to be a favorable approach.

Before detailing the impact of the Mulligan technique, it is necessary to clarify the effects that can be attributed to the therapeutic techniques administered to all groups included in the present study. For example, deep and superficial heaters utilized in traditional physiotherapy contribute to pain alleviation by diminishing muscle spasms. Moreover, they augment blood circulation through a vasodilatory impact on vessels and diminish the viscosity of viscoelastic collagen. This process enhances the resilience of soft tissues, making them less prone to strain during stretching [36]. Indeed, traditional stretching exercises and muscle-focused interventions have been shown to improve pain and functional scores [37]. Similarly, in our study, it was determined that significant improvement was achieved in terms of pain, disability and ROM in all 3 treatment groups in which stretching exercises, hot pack, TENS and ultrasonography were applied to the cervical region.

An interesting meta-analysis reported that placebo treatments had a moderate analgesic effect, possibly related to placebo effects that reduce pain through mechanisms independent of nociceptive processing [38]. Bizzarri et al. reported similar outcomes for single session thoracic manual therapy and single session placebo thoracic manual therapy in terms of pain among patients with shoulder dysfunction [39]. These effects can explain and support our findings concerning the level of improvement with the sham Mulligan technique and its superiority to the physiotherapy group.

The Mulligan techniques are based on the claim that biomechanical impairments may be associated with ’positional errors’ which cause the joint(s) to be misplaced [40]. There are results focusing on the effects of manipulation/mobilization techniques applied to the cervical region in the treatment of neck pain. Büyükturan et al. reported that cervical Mulligan SNAGs had significant benefits on pain, freedom of movement, functional level, kinesiophobia, depression and quality of life in older adults with neck pain [41]. In a study conducted by Vijayan et al. which included patients with nonspecific neck pain, it was found that short-term application of Mulligan SNAGs in combination with traditional physiotherapy had positive effects on pain, movement and function [11]. Similar results have been observed with Mulligan SNAGs in the short [42] and long term [43]. In a study comparing the Mulligan and Maitland mobilizations as well as conventional physiotherapy in neck pain, it was reported that both mobilization approaches created greater improvements in pain, disability and ROM [44]. There are also studies reporting no difference between these manual methods compared to other physical therapy methods [45]. In a prospective, randomized, single-blinded, prospective study by Shelke et al. comparing active cranio-cervical flexion (CCF) exercise (3 sets, 6–10 repetitions) and cervical Mulligan mobilization (3 sets, 6–10 repetitions), the results showed that both groups resulted similar levels of improvement [46]. Indifferent outcomes have also been shown by other studies comparing different techniques with ‘Mulligan concept’ therapies [47].

The study has some limitations. The fact that it was a single-center study limited the generalizability of its results. The most important limitation of this study is that post-intervention analyses were performed immediately after the last intervention, which prevents any interpretation of the sustained effects of the therapies. In each of the studies on Mulligan mobilization, there are different practices regarding the methodology and duration of treatment, and there is insufficient evidence as to which of these approaches are more valid and reliable. Another limitation of this study is that different duration and dose of treatment methods were not compared. The experience of the practitioner in treatments applied for neck pain could alter the impact of different treatments, and the study did not account for these variations. We also did not record analgesic drug use among participants during the intervention period; however, since the baseline pain levels were similar, the risk for biased outcomes due to this factor is minimal. Despite these limitations, the present study is valuable in terms of clearly demonstrating the utility of administering the upper thoracic Mulligan RNAGS technique for neck pain, and comparing the outcomes to recipients of physiotherapy only or sham Mulligan + physiotherapy.

## Conclusion

As a result of the analyses performed in the study, it can be said that physiotherapy applications together with Mulligan RNAGS mobilization applied to the upper thoracic region improve pain, ROM and functionality. It is also evident that the improvement obtained with the combined approach is superior to only physiotherapy and sham Mulligan. Our results may guide the choice of treatment techniques in the management of mechanical neck pain. In particular, we believe that the thoracic region should not be neglected in the treatment program, with potential applications involving spinal mobilization, static stretches and other physiotherapy techniques. However, there is a need to evaluate the medium- and long-term results of this therapy and to create guidelines describing treatment duration and methodology for specific situations.

## Supporting information

S1 Checklist CONSORT 2010 checklist of information to include when reporting a randomised trial*. (DOC)

S1 File (PDF)
