# Supplementary material for: Effects of upper thoracic Mulligan mobilization on pain, range of motion and function in patients with mechanical neck pain: A randomized placebo-controlled trial
Source: PLoS One. 2024 Oct 28;19(10):e0311206. doi: 10.1371/journal.pone.0311206 (PMC11516161; doi:10.1371/journal.pone.0311206)
Supplement: S1 File — (PDF) [file pone.0311206.s002.pdf]

SİVAS CUMHURİYET UNIVERSITY
NON-INTERVENTIONAL CLINICAL RESEARCH ETHICS COMMITTEE
APPLICATION FORM

**Application date and no: 02.12.2020 / 2020-12-13**
**Decision date and no: 16.12.2020 / 2020-12-13**
**Title:** Effects of Upper Thoracic Mulligan Mobilization for Pain, Range of Motion and Function in Patients with Mechanical Neck Pain

Have you applied to another ethics committee for the same project before?

YES ☐ **NO** ☒

A-GENERAL INFORMATION:

1. Research coordinator

Name-surname and title: Assist. Prof. Dr. Ömer Osman PALA

Signature

Institution and address: Sivas Cumhuriyet University, Faculty of Health Sciences, Department of Physiotherapy and Rehabilitation

Phone: 0(532) 797 57 16

E-mail: fzt.omerpala@gmail.com

2- Other researchers involved in the research:

Name Surname, Title	Department or Division	Signature
Ramazan ÇEVİK	Physiotherapy and Rehabilitation	

3- Name, title, institutional identity of the persons who will provide technical support to the research other than co-investigators

4- The unit where the research will be conducted: Mengücek Gazi Training and Research Hospital Physiotherapy Unit

5- Within the scope of the study, is it planned to send biological material to another institution in Turkey or abroad?
YES ☐ **NO** ☒

ATTENTION! If your answer is YES, the Biological Material Transfer Agreement must be completed.

B- INFORMATION ABOUT THE RESEARCH

1-Type of research:
☒ Master's, doctorate or specialisation thesis
☐ Academic research
☐ Other: ...

2. Nature of the research:

☐ Studies to be carried out with biochemistry, microbiology, pathology and radiology collection materials such as blood, urine, tissue, images or materials obtained during routine examinations, tests, analyses and treatment procedures

☒ Research to examine the effect of rehabilitation programs such as diet and physiotherapy

☐ If the scale will be used in survey-based studies;

- ☐ **Published by the author. No permission from the author is required.**
- ☐ **Not published by the author. Permission has been obtained from the author.**

☐ Retrospective archive searches and similar observational studies using file and image records

☐ Other (Explain briefly.)

☐ Observational studies (except observational medical device and observational drug studies)

☐ Cell or tissue culture studies

☐ Research with genetic material for identification purposes, excluding gene therapy clinical trials

☐ Research to be conducted within the boundaries of nursing activities

☐ Dietary studies with food additives

☐ Research on body physiology such as exercise

☐ Studies based on anthropometric measurements

☐ All research that does not require the direct intervention of a physician, such as research on the assessment of living habits

☐ Retrospective archive searches and similar observational studies using file and image records

3. Centres Participating in the Study:

☒ One centre

☐ Single centre-Multidisciplinary

☐ Very centre

4. a. Research Coordinator in Multicenter or Multidisciplinary Research:

Name.	Surname	Title	Address

4. b. External Centers and Responsible Persons Referred to in Multicenter Studies:

Name.	Surname	Title	Address

5- The objective, significance and scientific background of the research:

With technology taking an important place in our lives, problems related to the musculoskeletal system have started to increase in people (1). Neck pain, which is one of the musculoskeletal system problems, is estimated to be experienced by 70% of people at certain periods of their lives (2). It is estimated that 25% of the people who apply to outpatient physiotherapy departments of hospitals are due to neck pain (3-4). 78% of men and 85% of women who experience neck pain for different periods of time report that their symptoms never disappear (5). The most important features of mechanical neck pain (MBA), which is

characterized by pain, limitation in joint movement and functional limitation (6). MBA may be caused by problems in ligaments, muscles, intervertebral discs and vertebral joints (7).

When the studies investigating the causes of MBA and the efficacy of different treatment methods are analysed, there is no method accepted as the gold standard for the treatment of MBA (8-9-10). One of the preferred treatment options for MBA is physiotherapy and rehabilitation. Physiotherapy and rehabilitation includes electrotherapy modalities, therapeutic exercises, joint mobilisation and manipulations, and myofascial release. Among these treatments, manual therapy, which targets the harmony of structure and function, is the most preferred treatment method by physiotherapists (11-12). Manual therapy provides improvement by orienting the body's biomechanics, circulation and body structure by directly affecting the joints, muscles and soft tissues (13). In the literature, there are studies on both cervical mobilisation and thoracal mobilisation for neck pain. Cervical mobilisation and manipulation in neck pain are controversial because they carry certain risks (14). In one study, it was reported that mobilisation of the thoracal spine decreased pain in the cervical region and increased mobility in this region (16). Cho et al. (2017) reported that the effect of thoracal spine mobilisation on pain and range of motion was greater than the effect of cervical spine mobilisation in their study on individuals with neck pain (17). There are different concepts such as Mckenzi, Kalterrnborn, Mulligan and Maitland in manual therapy (10). Mulligan method includes mobilisation of the joints in a certain position with active movement within the framework of certain principles and aims to eliminate musculoskeletal pain and movement limitation (18).

When the literature was examined, there was no study examining the effect of the Mulligan concept natural apophyseal reversal technique on MBA. This study was planned to investigate the effect of natural apophyseal reversal on pain and movement limitation in individuals diagnosed with MBA.

6-Research protocol, materials and methods:

<u>Individuals</u>

In the calculation of the sample group, it was found that there should be 69 participants for a power of 0.90 at a significance level of 0.05 using the G*Power programme. The study will be conducted in Erzincan Mengücek Gazi Training and Research Hospital Physical Therapy and Rehabilitation Unit. The 69 participants will be divided into three groups: Mulligan group (MG), Sham (different direction and amplitude of mobilisation) group (SG) and traditional physiotherapy group (FG). An online randomisation tool "Graph Pad" will be used to determine the groups and stratified randomisation will be applied to ensure equal numbers of men and women. Individuals will be treated for 11 sessions (every day for the first week and every other day for the next two weeks).

Inclusion Criteria:
Those between the ages of 18-65
Those with neck pain lasting at least 3 months
Individuals diagnosed with mechanical neck pain by a specialist physician

Exclusion Criteria:
Those with a history of spinal surgery
Those with a history of whiplash injury
Those with a history of trauma in the cervical and thoracic region
Pregnancy

Interventions

Experimental: Mulligan group
Mulligan Reverse Natural Apophysial Glide Technique (RNAGS)
Mulligan mobilization techniques was applied to the upper thoracic segments with the RNAGS technique. The application was performed with one hand (thumb in extension, other fingers flexed) that was used to perform the pushing maneuver on the transverse processes, while the other arm was used to gently grasp the head of the patient and recline the neck against the body for stabilization.

Individuals underwent 11 sessions (every day for the first week, every other day for the next two weeks).Hot pack was applied to the neck and upper trapezius area for 20 minutes.2 channels and 4 electrodes in the neck area; Conventional transcutaneous electrical nerve stimulation (TENS) was applied to the patient for 20 minutes with current transition time: 50-100 microseconds, frequency: 60-120 Hz and mild tingling without causing discomfort.US application was performed at a frequency of 1.5 W/cm2 and 1 Mhz for 8 minutes. Stretching exercises were applied to the trapezius upper part and levator scapula muscles for 15-30 seconds with 10 repetitions under the supervision of a physiotherapist. In addition to physiotherapy applications in MG, Mulligan natural apophyseal reversal technique was applied to the upper thoracic segments. Mulligan mobilization was performed with 3 sets of 10 repetitions and 15-20 seconds of rest between sets.

Sham (different in the direction and amplitude of mobilization) group
A sham Mulligan RNAGS technique with low pressure compression was applied to thoracic region. Individuals underwent 11 sessions (every day for the first week, every other day for the next two weeks).Hot pack was applied to the neck and upper trapezius area for 20 minutes.2 channels and 4 electrodes in the neck area; Conventional TENS was applied to the patient for 20 minutes with current transition time: 50-100 microseconds, frequency: 60-120 Hz and mild tingling without causing discomfort.US application was performed at a frequency of 1.5 W/cm2 and 1 Mhz for 8 minutes.Stretching exercises were applied to the trapezius upper part and levator scapula muscles for 15-30 seconds with 10 repetitions under the supervision of a physiotherapist.Sham mobilization was applied to the segments where Mulligan mobilization was carried out in Sham group, in which the direction of thrust and thrust were different.

Conventional physical therapy group
The same physiotherapy programme will be applied to all three groups. Conventional TENS was applied to the neck area with 2 channels and 4 electrodes for 20 min (current passage

time: 50-100 microseconds, frequency: 0-120 Hz). Ultrasonography was performed for 8 minutes with an intensity of 1.5 w/cm2 and a frequency of 1 MHz. Stretching exercises were applied to the upper part of the trapezius and levator scapula muscles for 15-30 seconds for 10 repetitions by a physiotherapist

Outcome Measures

Range of Motion Assessment
Active flexion, extension, right and left lateral flexion and rotations of the cervical region will be measured while the individuals are in the sitting position. The DrGonyometer (ios) application, which has a reliability study for cervical range of motion measurements, will be used (19).

Pain Assessment
The pain intensity assessment of individuals will be performed with the Visual Analogue Scale (VAS) (20).Individuals are asked to mark the pain they feel on a 10cm long (VAS) scale. "0" indicates that the pain is not felt at all and "10" indicates unbearable pain (21).

Assessment of Neck Disability
The neck disability status of the individuals will be evaluated with the Neck Disability Index (NDI)(22). This index, which consists of 10 items with proven reliability and validity, evaluates subjective symptoms and activities of daily living. Each answer is scored between 0 (no limitation) and 5 (maximum limitation). In the evaluation of the scoring, a score between 0 and 4 is interpreted as no disability, a score between 5 and 14 as mild disability, a score between 15 and 24 as moderate disability, a score between 25 and 34 as severe disability and a score between 35 and 50 as the worst functional status (23).

Statistical Analysis
SPSS® (Statistical Package for Social Sciences) version 22 will be used to analyse the data obtained from the study and to create tables. Mean and standard deviation values will be used for continuous variables (quantitative) and frequency and percentage values will be used for categorical variables (qualitative). Kolmogorov Smirnov test, skewness kurtosis index and graphical methods will be used to investigate the conformity of quantitative variables to normal distribution. In the comparison of differences between groups, One-Way Analysis of Variance will be used for normally distributed variables and Kruskal Wallis-H test will be used for non-normally distributed variables. Whether the change in the measurements taken at different times within each group is significant or not will be analysed by t-test in Dependent Sample for normally distributed variables and Wilcoxon test for non-normally distributed variables. In all statistical analyses, $p<0.05$ will be accepted as significance level.

7. Research Team and Project Specific Tasks:

a) Principal Investigator::

Name.	Surname	Title	Project Responsibility	Signature
Ömer Osman	PALA	Assist. Prof. Dr.	Planning the study<br>Formal analysis<br>Methodology<br>Project administration<br>Supervision<br>Validation<br>Visualization<br>Writing – review & editing	

b) Co-researchers:

Name.	Surname	Title	Project Responsibility	Signature
Ramazan	ÇEVİK	PT	Data curation<br>Physiotherapy applications<br>Evaluation of cases<br>Formal analysis<br>Investigation<br>Methodology<br>Resources<br>Writing – original draft	

8-Targeted start and end date and duration of the research:

1 January 2021 - 30 May 2021

9- Possible sponsor and budget of the research:

To be covered by the principal investigator (279 TL)
DrGoniometer smartphone app fee: 79 TL
Stationery, photocopy and file costs: 200 TL

10- References

1.Lee, S., Lee, D.,Park, J. (2015). Effect of the cervical fexion angle during smart phone use on muscle fatigue of the cervical erector spinae and upper trapezius. *Journal of physical therapy science*, 27 (6), 1847-1849.

2.Palmer KT., Walsh K., Bendall H. (2000). Back pain in Britain: comparison of two prevalence surveys at an interval of 10 years. *BMJ*, 320:1577–1578.

3.Jette AM, Smith K, Haley SM. (1994). Physical therapy episodes of care for patients with low back pain. *Phys Ther*, 74: 101–110. [Medline]

4.Gummesson C, Isacsson SO, Isacsson AH. (2006). The transition of reported pain in different body regions: a one-year follow-up study. *BMC Musculoskelet Disord*, 7: 17. [Medline] [CrossRef]

5.Pernold G, Mortimer M, Wiktorin C. (2005). Neck/shoulder disorders in a general population: natural course and influence of physical exercise—a 5-year follow-up. *Spine*, 30:E363–E368.

6.Barton, P.M., Hayes, K.C. (1996). Neck flexor muscle strength, efficiency, and relaxation times in normal subjects and subjects with unilateral neck pain and headache. *Archives of physical medicine and rehabilitation,* 680-687.71,

7.Maitland GD, Hengeveld E, Banks K. (2001). Maitland's Vertebral Manipulation. *6th ed. Oxford: Butterworth-Heinemann,* pp 93–324

8.Hudson JS, Ryan CG. (2010). Multimodal group rehabilitation compared to usual care for patients with chronic neck pain: a pilot study. *Man Ther*, 15: 552–556. [Medline] [CrossRef]

9.Hoving JL, Gross AR, Gasner D. (2001). A critical appraisal of review articles on the effectiveness of conservative treatment for neck pain. *Spine*, 26:196 –205.

10.Gross AR, Aker PD, Goldsmith CH. (2000). Peloso P. Patient education for mechanical neck disorders. *Cochrane Database Syst Rev*, (2):CD000962

11.Childs JD, Cleland JA, Elliott JM. (2008). Neck pain: a clinical practice guideline linked to the International Classification of Functioning, Disability, and Health from the Orthopaedic Section of the American Physical Therapy Association. *J Orthop Sports Phys Ther*, 38:A1-A34.

12.Bryans R, Decina P, Descarreaux M. (2014). Evidence-based guidelines for the chiropractic treatment of adults with neck pain. *J Manipulative Physiol Ther*, 37:42-63.

13.Korr IM. (1975). Proprioceptors and somatic dysfunction.*The Journal of the American Osteopathic Association,* 74(7):638-50

14.Puentedura EJ, March J, Anders J. (2012). Safety of cervical spine manipulation: are adverse events preventable and are manipulations being performed appropriately? A review of 134 case reports. *J Man Manip Ther*, 20: 66-74.

15.Krauss J, Creighton D, Ely JD. (2008). The immediate effects of upper thoracic translatoric spinal manipulation on cervical pain and range of motion: a randomized clinical trial. *J Manual Manip Ther*, 16: 93–99. [Medline] [CrossRef

16.Cho, J., Lee, E., & Lee, S. (2017). Upper thoracic spine mobilization and mobility exercise versus upper cervical spine mobilization and stabilization exercise in individuals with forward head posture: a randomized clinical trial. *BMC musculoskeletal disorders*, *18*(1), 1-10.

17.Giles, L. (1997). Clinical anatomy and management of low back pain: LGF Giles and KP Singer(ed.) (c. 1). Oxford: Butterworth Heinemann.

18.Miller, J. (1999). The Mulligan Concept–the next step in the evolution of manual therapy. *Canadian Physiotherapy Association Orthopaedic Division Review*, March/April, 9-13

19.Hales, G., Keating, R., Bear, N., Warren, K., Otter, S. (2015). Reliability of a smartphone goniometer app compared with traditional goniometer for measuring passive motion at the first metatarsophalangeal joint. *J Foot Ankle Res*, 8(2):2-12

20.Jensen MP, Turner JA, Romano JM, Fisher LD. (1999). Comparative reliability and validity of chronic pain intensity measures. *Pain*, 83(2):157-62.

21.Katz J, Melzack R. (1999). Measurement of pain. *Surg Clin North Am*, 79(2):231-52.

22.Vernon H, Mior S. (1991). The Neck Disability Index: a study of reliability and validity. *J Manipulative Physiol Ther,* 14(7):409-15.

23.Aslan E, Karaduman A, Yakut Y, Aras B, Simsek IE, Yagly N. (2008). The cultural adaptation, reliability and validity of neck disability index in patients with neck pain: a Turkish version study. *Spine* (Phila Pa 1976), 33(11):E362-5.
